# Predicting response to neoadjuvant therapy in breast cancer using longitudinal DCE-MRI deep learning integrated with tumor microenvironment data

**DOI:** 10.3389/fimmu.2026.1749668

**Published:** 2026-04-28

**Authors:** Lan Yan, Xianming Huang, Lan Liu, Ao Wu, Yingyi Luo, Hao Li, Shaofeng Yi, Tenghua Yu, Qiao Zeng

**Affiliations:** 1 Department of Radiology, Jiangxi Cancer Hospital & Institute, Jiangxi Clinical Research Center for Cancer, The Second Affiliated Hospital of Nanchang Medical College, Nanchang, China; 2 Department of Pathology, Jiangxi Cancer Hospital & Institute, Jiangxi Clinical Research Center for Cancer, The Second Affiliated Hospital of Nanchang Medical College, Nanchang, China; 3 Jiangxi Medical College, Nanchang University, Nanchang, China; 4 Department of Breast Surgery, Jiangxi Cancer Hospital & Institute, Jiangxi Clinical Research Center for Cancer, The Second Affiliated Hospital of Nanchang Medical College, Nanchang, China; 5 Department of Radiology, Jiangxi Cancer Hospital & Institute, Jiangxi Clinical Research Center for Cancer, The Second Affiliated Hospital of Nanchang Medical College, JXHC Key Laboratory of Tumour Metastasis (Jiangxi Cancer Hospital), Nanchang, China

**Keywords:** breast cancer, deep learning, dynamic contrast-enhanced MRI, neoadjuvant therapy, pathological complete response, tumor microenvironment, tumor-infiltrating lymphocytes

## Abstract

**Objective:**

This study developed and validated a multimodal fusion model to enable the early and accurate prediction of pathological complete response (pCR) to neoadjuvant therapy (NAT) in breast cancer. The model integrates deep learning (DL) features derived from longitudinal dynamic contrast-enhanced magnetic resonance imaging (DCE-MRI) acquired early during treatment, peripheral blood inflammatory (PBI) indices, and baseline levels of tumor-infiltrating lymphocytes (TILs).

**Methods:**

A total of 262 breast cancer patients receiving NAT were retrospectively enrolled and divided into a training cohort (n=183) and a validation cohort (n=79) based on the time of surgery. Deep learning models (Pre-NAT DL and Post-2nd-NAT DL) were constructed using features extracted from pre-treatment (baseline) and post-second-cycle DCE-MRI images, respectively. An immune-inflammation model was built using baseline TILs and dynamically changing PBI indices. A clinical model was developed based on baseline clinicopathological characteristics. Finally, a combined model was constructed by integrating features from all the aforementioned modalities. The models were developed using various machine learning algorithms, and their predictive performance was assessed and compared.

**Results:**

In the validation cohort, the combined model achieved superior predictive performance, with an area under the receiver operating characteristic curve of 0.90 and specificity of 95%. Its performance was significantly better than that of any single-modality model. The Post-2nd-NAT DL model (AUC = 0.85) outperformed the Pre-NAT DL model (AUC = 0.75), confirming the critical predictive value of deep learning features from early-treatment DCE-MRI. The immune-inflammation model also exhibited independent predictive capability (AUC = 0.73).

**Conclusion:**

The combined model integrating deep learning features from early longitudinal DCE-MRI, dynamic systemic inflammatory indicators, and baseline TILs significantly enhances the early prediction of pCR to NAT in breast cancer. This multimodal fusion strategy offers a potential tool to aid personalized treatment planning in breast cancer patients undergoing NAT.

## Introduction

Neoadjuvant therapy (NAT) is a standard treatment for patients with locally advanced breast cancer, and its efficacy directly influences surgical decisions and patient prognosis (1). The accurate and early prediction of treatment response is critical for optimizing individualized regimens, thereby avoiding ineffective therapies and unnecessary toxicity (2). However, there is currently a lack of reliable non-invasive biomarkers in clinical practice for reliably predicting efficacy in the early stages of treatment. This limitation has spurred the development of multimodal, cross-scale predictive models (3).

Our team’s previous research has demonstrated that radiomic features based on dynamic contrast-enhanced magnetic resonance imaging (DCE-MRI) show significant potential for the early prediction of NAT response (4–6). Radiomics, by extracting and analyzing a high volume of quantitative features from medical images, can reveal intratumoral heterogeneity (7). However, conventional radiomics relies on handcrafted features, which have limited representational capacity, thus restricting its broader clinical application. Deep learning (DL) techniques can automatically learn hierarchical feature representations from data, providing a distinct advantage in capturing lesion complexity and dynamic changes. This capability offers a promising technical pathway for significantly improving prediction accuracy (8).

In recent years, the role of the tumor microenvironment (TME) in tumor initiation, progression, and treatment response has gained increasing attention (9). The TME is a complex ecosystem composed of tumor cells, immune cells, stromal cells, vascular networks, and various signaling molecules. Among these, tumor-infiltrating lymphocytes (TILs), as a core component of the TME, are key effector cells in anti-tumor immune responses and are strongly associated with prognosis and survival, particularly in triple-negative breast cancer (TNBC) and HER2-positive breast cancer (10, 11). TILs have also been confirmed to be significantly correlated with the rate of pathological complete response (pCR) to neoadjuvant therapy in breast cancer (12). On the other hand, the systemic inflammatory response represents another critical dimension of the TME, which can be quantified using peripheral blood inflammatory (PBI) indices such as the Neutrophil-to-Lymphocyte Ratio (NLR), Platelet-to-Lymphocyte Ratio (PLR), and Systemic Immune-inflammation Index (SII) (13). These indices reflect the balance of the body’s immune and inflammatory status and play important roles in tumor progression, metastasis, and treatment resistance. However, the joint influence of TILs and systemic inflammation on treatment efficacy, and their potential complementary value to deep learning features, remain poorly understood.

Therefore, this study aims to integrate DL features extracted from longitudinal DCE-MRI images acquired early during treatment, PBI indices, and baseline TILs levels. The objective is to construct a multi-parameter, fused predictive model for the non-invasive and accurate assessment of treatment response in breast cancer patients during the early courses of NAT. This integrated imaging-immune-inflammatory approach seeks to provide a more reliable basis for individualized clinical treatment decision-making.

## Methods

### Study population

**Figure 1:**
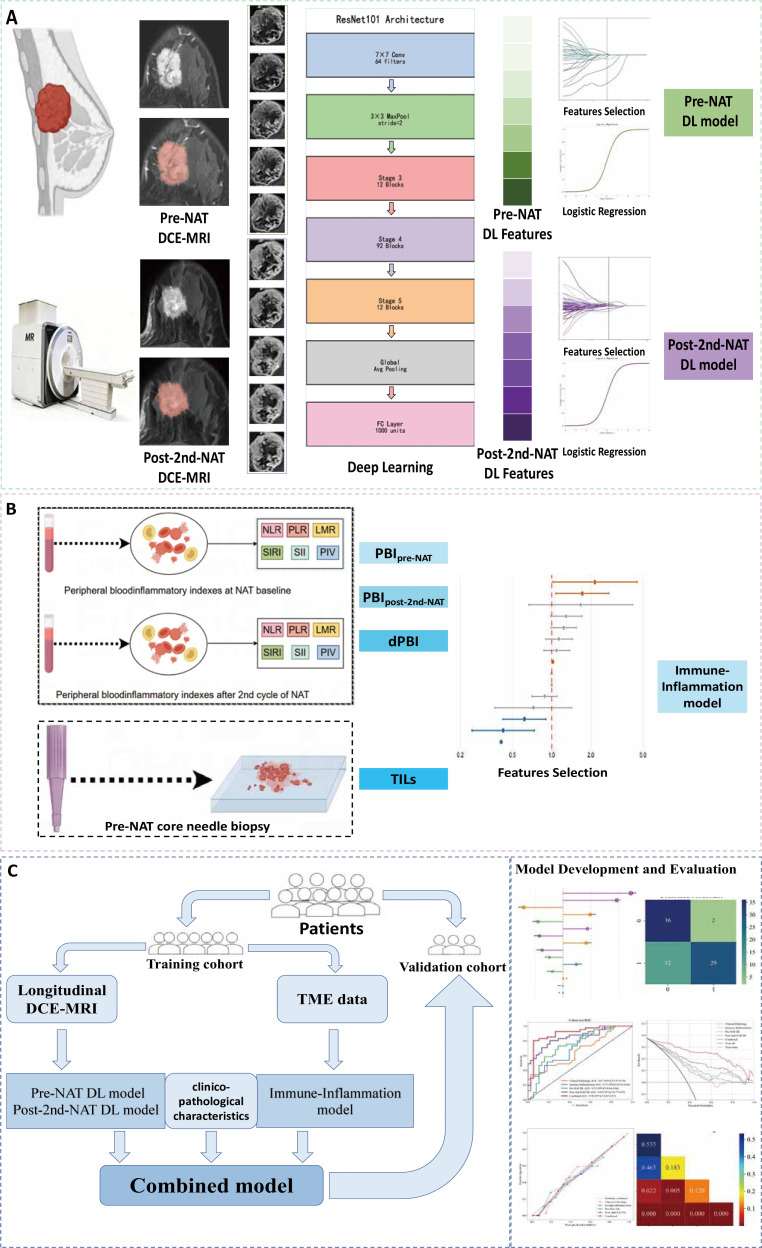
Study workflow for the development and validation of predictive models. The schematic illustrates the sequential pipeline for building multi-modal models to predict treatment response in breast cancer. **(A)** Development of deep learning (DL) models from dynamic contrast-enhanced magnetic resonance imaging (DCE-MRI) acquired pre- and post-neoadjuvant therapy (NAT). **(B)** Construction of an immune-inflammation model from peripheral blood inflammatory (PBI) indices and baseline tumor-infiltrating lymphocytes (TILs). **(C)** Integration of models and comprehensive evaluation to identify the optimal predictor.

This retrospective study follows the detailed flowchart depicted in **Figure 1**. This retrospective study enrolled breast cancer patients who received NAT followed by surgical resection between October 2019 and April 2025. The inclusion criteria were as follows (1): Pathological confirmation of breast cancer by core needle biopsy at baseline before treatment (2); Completion of 4–8 cycles of neoadjuvant therapy (3); Undergoing surgical resection after NAT (4); Availability of DCE-MRI scans and relevant laboratory tests performed at baseline and after the 2nd cycle. The exclusion criteria were: (1) Missing imaging, clinical, or pathological data; (2) History of malignant tumors at other sites; (3) Presence of active infection at baseline or after the 2nd cycle of NAT. This study was approved by the institutional review board of our hospital (Approval No.: 2025ky117). The requirement for informed consent was waived due to the retrospective nature of the study. Patients were divided into a training cohort (70%, surgeries prior to December 25, 2022) and a validation cohort (30%, surgeries following this date) to ensure temporal validation. Clinical data such as patient sex, age, clinical stage, tumor markers carbohydrate antigen 125 (CA125), CA153, and carcinoembryonic antigen (CEA) were also collected.

### Definition and data collection of PBI indices

Peripheral blood laboratory data were extracted from medical records at baseline and after the second treatment cycle (within one week before the first and third cycle, respectively). Collected parameters included neutrophil count (×10^9^/L, N), monocyte count (×10^9^/L, M), platelet count (×10^9^/L, P), lymphocyte count (×10^9^/L, L), and albumin concentration (g/L, A). The following inflammatory indices were calculated: neutrophil-to-lymphocyte ratio (NLR) = N/L, monocyte-to-lymphocyte ratio (MLR) = M/L, platelet-to-lymphocyte ratio (PLR) = P/L, systemic immune-inflammation index (SII) = P × NLR, systemic inflammation response index (SIRI) = N × MLR, and pan-immune-inflammation value (PIV) = (N × M × P)/L (13). Dynamic peripheral blood inflammatory (dPBI) indices were defined as the post-treatment value minus the baseline value (e.g., dNLR = NLR_post-2nd-NAT_ − NLR_baseline_).

### Pathological evaluation

Immunohistochemistry (IHC) was performed on all biopsy and surgical specimens. HER2 positivity was defined as an IHC result of 3+, or IHC 2+ with confirmed HER2 gene amplification by FISH. The positivity thresholds for ER and PR were set at ≥1% of tumor cells showing positive nuclear staining. Stromal TILs were assessed on hematoxylin and eosin (H&E)-stained sections according to the guidelines of the International TILs Working Group (14, 15). After formalin fixation and paraffin embedding of the surgical resection specimens from all enrolled cases, H&E-stained sections were prepared. The evaluation specifically targeted the stromal TILs excluding areas within tumor cell nests and regions of necrosis. Initially, the stromal areas were identified under low-power magnification (×40–×100) to preliminarily determine the type of inflammatory cell infiltration. Subsequently, the percentage of the stromal area occupied by mononuclear inflammatory cells (including lymphocytes and plasma cells) was quantitatively assessed under high-power fields (×200–×400). Based on international standards, cases were categorized into the low-TILs group (TILs <10%) and the high-TILs group (TILs ≥10%) (16). All slides were independently evaluated by a designated, experienced pathologist who was blinded to the clinical and imaging information of the patients. In cases where assessment was ambiguous or borderline, a second pathologist was consulted for review, and a final score was determined by consensus. pCR was defined as the absence of residual invasive carcinoma in both the breast primary site and axillary lymph nodes (ypT0/is, ypN0), with or without the presence of ductal carcinoma *in situ* (17).

### Magnetic resonance imaging and deep learning

#### Image acquisition and preprocessing

All images were retrieved from the Picture Archiving and Communication System (PACS). Detailed dynamic contrast enhanced-MRI (DCE-MRI) acquisition parameters are provided in **Supplementary Material**. The study utilized images from the peak enhancement phase of dynamic contrast-enhanced MRI. To minimize variations from different protocols, all images were resampled to an isotropic 1×1×1 mm³ resolution using B-spline interpolation and underwent intensity normalization via Z-score standardization (17). The delineation region of interest (ROI) for breast tumor was primarily performed by an attending radiologist with over ten years of experience in breast imaging. Detailed information regarding the tumor segmentation process is described in **Supplementary Material**.

### Deep transfer learning model

We used a pre-trained ResNet-101 model, initialized with ImageNet weights and implemented in PyTorch (18, 19). Inputs were built from standardized and resampled DCE-MRI ROIs mentioned above. A 2.5D format was created by stacking the largest tumor slice along with two slices above and two slices below (five slices in total). The model was fine-tuned using transfer learning. First, all convolutional layers were frozen while only the new classifier (with dropout set to 0.5) was trained. Then, the entire network was unfrozen for end-to-end fine-tuning. Training was conducted with the following hyperparameters: optimizer: AdamW; learning rate: 1×10^-4^ (classifier) and 5×10^-5^ (full network); batch size: 16; max epochs: 50 (20). We applied weighted binary cross-entropy loss to handle class imbalance and used early stopping (patience: 10 epochs). Data augmentation included random horizontal flipping, ± 10° rotation, and ±10% brightness/contrast adjustment. More details regarding the DL model are provided in the **Supplementary Material**. Ultimately, using the ResNet-101 model, 2048 deep learning features were extracted from the baseline and post-2nd-NAT DCE-MRI ROIs, respectively. All experiments ran on an NVIDIA GeForce RTX 4080 SUPER GPU.

To make the decision-making process of the model more transparent and investigate its interpretability, we employ Gradient-weighted class activation mapping (Grad-CAM) to visualize the ResNet-101 model, aiming to reveal the image regions the model focuses on when making pCR predictions (21).

### Model development and evaluation

To predict pCR following NAT, this study developed the following models (1): Pre-NAT DL model: developed using the 2.5D DL features derived from pre-treatment DCE-MRI images, with feature selection performed via T-test, Pearson correlation analysis, and Least Absolute Shrinkage and Selection Operator (LASSO) regression. (2) Post-2nd-NAT DL model: built using the 2.5D DL features from post-2nd-NAT DCE-MRI images, following the same feature selection procedure. (3) Immune-Inflammation model: constructed by selecting PBI indices (measured at baseline, 2nd-cycle, and dPBI) and baseline TILs levels that demonstrated statistically significant differences between the pCR and non-pCR groups. (4) Clinical-Pathology model: built using baseline clinicpathological characteristics that showed statistically significant differences between the pCR and non-pCR groups. (5) Combined model: developed by pooling variables from all the above models and performing stepwise regression with minimum Akaike Information Criterion (AIC) for final variable selection.

All models were developed using various machine learning algorithms, including logistic regression (LR), naive Bayes (NB), support vector machine (SVM), k-nearest neighbors (KNN), random forest (RF), extreme gradient boosting (XGBoost), adaptive boosting (AdaBoost), and multilayer perceptron (MLP). The best-performing algorithm for each model in the validation set was selected as the final model. Model performance was evaluated and compared using the area under the receiver operating characteristic (ROC) curve (AUC), accuracy, sensitivity, specificity, positive predictive value (PPV), negative predictive value (NPV), F1-score, and DeLong test.

### Statistical analysis

Statistical analyses in this study were performed using Python (version 3.9.5) and R (version 4.3.1). Continuous variables are presented as median (interquartile range), and comparisons between groups were conducted using the T test for normally distributed data or the Mann-Whitney U test for non-normally distributed data, based on the results of the Shapiro-Wilk normality test. Categorical variables are described as frequencies (percentages), and group comparisons were made using the Chi-squared test or Fisher’s exact test, as appropriate. A two-sided P-value < 0.05 was considered statistically significant.

## Results

### Patients

This study enrolled a total of 262 breast cancer patients (median age, 50 years [IQR, 47–52 years]) who received NAT. Pathological complete response was achieved in 37.7% (69/183) and 51.9% (41/79) of patients in the training and validation cohorts, respectively. The characteristics of all patients, including age, tumor location, clinical stage, tumor markers (CEA, CA153, CA125), hormone receptor status (ER, PR), HER2 status, Ki-67 index, and PBI indicators, are summarized in **Table 1**.

**Table 1:** Characteristics of the study population in the training and validation cohorts.

Characteristics	Training cohort (n = 183)	Validation cohort (n = 79)	Total (n = 262)	P value
Treatment Response				0.033
Non-pCR	114 (62.30%)	38 (48.10%)	152 (58.02%)	
pCR	69 (37.70%)	41 (51.90%)	110 (41.98%)	
Age	50.00 (47.00, 52.00)	50.00 (47.00, 52.00)	50.00 (47.00, 52.00)	0.732
Location				0.076
Left	87 (47.54%)	47 (59.49%)	134 (51.15%)	
Right	96 (52.46%)	32 (40.51%)	128 (48.85%)	
Clinical stage				0.728
I	4 (2.19%)	2 (2.53%)	6 (2.29%)	
II	72 (39.34%)	31 (39.24%)	103 (39.31%)	
III	96 (52.46%)	44 (55.70%)	140 (53.44%)	
IV	11 (6.01%)	2 (2.53%)	13 (4.96%)	
ER				0.330
negative	79 (43.17%)	29 (36.71%)	108 (41.22%)	
positive	104 (56.83%)	50 (63.29%)	154 (58.78%)	
PR				0.998
negative	88 (48.09)	38 (48.10%)	126 (48.09%)	
positive	95 (51.91%)	41 (51.90%)	136 (51.91%)	
HER2				0.812
negative	99 (54.10%)	44 (55.70%)	143 (54.58%)	
positive	84 (45.90%)	35 (44.30%)	119 (45.42%)	
Ki67	50.00 (30.00, 70.00)	50.00 (30.00, 70.00)	50.00 (30.00, 70.00)	0.598
TILs				0.414
Low	53 (28.96%)	19 (24.05%)	72 (27.48%)	
High	130 (71.04%)	60 (75.95%)	190 (72.52%)	
CEA	1.58 (0.96, 2.71)	1.42 (0.80, 2.87)	1.50 (0.91, 2.71)	0.461
CA153	10.60 (7.55, 17.80)	10.62 (7.40, 18.85)	10.61 (7.53, 18.38)	0.671
CA125	14.26 (10.72, 23.02)	13.12 (9.57, 18.36)	13.68 (10.25, 21.01)	0.140
NLR_pre-NAT_	2.27 (1.79, 3.26)	1.95 (1.62, 2.83)	2.18 (1.74, 3.10)	0.061
PLR_pre-NAT_	144.97 (118.87, 189.06)	150.00 (114.86, 186.26)	145.95 (117.42, 188.30)	0.919
LMR_pre-NAT_	5.07 (3.94, 6.71)	5.23 (3.97, 6.52)	5.09 (3.95, 6.67)	0.744
SIRI_pre-NAT_	0.73 (0.48, 1.11)	0.68 (0.47, 0.83)	0.70 (0.48, 1.05)	0.543
SII_pre-NAT_	536.95 (365.24, 850.93)	490.38 (390.57, 671.48)	515.50 (373.33, 772.88)	0.372
PIV_pre-NAT_	161.90 (105.31, 286.26)	153.09 (111.67, 231.15)	160.75 (105.70, 276.56)	0.977
NLR_post-2nd-NAT_	2.52 (1.71, 3.73)	2.09 (1.46, 3.17)	2.40 (1.59, 3.57)	0.062
PLR_post-2nd-NAT_	204.97 (149.31, 299.38)	194.05 (134.87, 290.44)	202.56 (144.53, 298.05)	0.420
LMR_post-2nd-NAT_	3.12 (2.38, 4.77)	3.65 (2.67, 5.16)	3.35 (2.46, 4.84)	0.106
SIRI_post-2nd-NAT_	0.97 (0.57, 1.67)	0.70 (0.39, 1.44)	0.93 (0.50, 1.58)	0.032
SII_post-2nd-NAT_	656.37 (392.26, 1077.74)	516.82 (318.97, 942.72)	617.88 (354.90, 1053.56)	0.083
PIV_post-2nd-NAT_	249.01 (123.35, 498.40)	179.69 (76.44, 398.37)	226.32 (104.19, 480.30)	0.062
dNLR	0.23 (-0.74, 1.29)	-0.04 (-0.92, 0.92)	0.18 (-0.78, 1.24)	0.261
dPLR	61.21 (3.34, 139.88)	19.21 (-18.52, 115.12)	48.26 (-3.58, 131.35)	0.015
dLMR	-1.81 (-2.92, -0.37)	-1.31 (-2.92, 0.52)	-1.60 (-2.92, -0.07)	0.168
dSIRI	0.25 (-0.21, 0.85)	0.06 (-0.46, 0.71)	0.18 (-0.27, 0.81)	0.055
dSII	116.72 (-178.75, 555.28)	37.32 (-223.02, 289.93)	85.70 (-196.86, 494.90)	0.130
dPIV	79.86 (-44.98, 269.13)	21.02 (-105.26, 223.80)	50.86 (-68.16, 256.42)	0.062

Data are presented as n (%) for categorical variables and median (interquartile range) for continuous variables. pCR, pathological complete response; Non-pCR, non-pathological complete response; ER, Estrogen Receptor; PR, Progesterone Receptor; HER2, Human Epidermal Growth Factor Receptor 2; Ki-67, marker of proliferation Ki-67; TILs, Tumor-Infiltrating Lymphocytes; CEA, Carcinoembryonic Antigen; CA153, Carbohydrate Antigen 153; CA125, Carbohydrate Antigen 125; NLR, Neutrophil-to-Lymphocyte Ratio; PLR, Platelet-to-Lymphocyte Ratio; LMR, Lymphocyte-to-Monocyte Ratio; SIRI, Systemic Inflammation Response Index; SII, Systemic Immune-inflammation Index; PIV, Pan-immune-inflammation Value; d, delta (change).

### Model development

**Figure 2:**
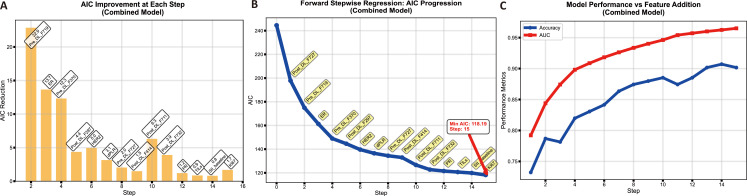
Akaike Information Criterion (AIC) based model selection process for the combined model. **(A)** Bar chart showing the AIC reduction achieved at each step of the feature selection process. **(B)** Line chart illustrating the progression of the AIC value during the forward stepwise regression. **(C)** Line chart comparing the model performance metrics (AUC and Accuracy) against the number of features added.

**Figure 3:**
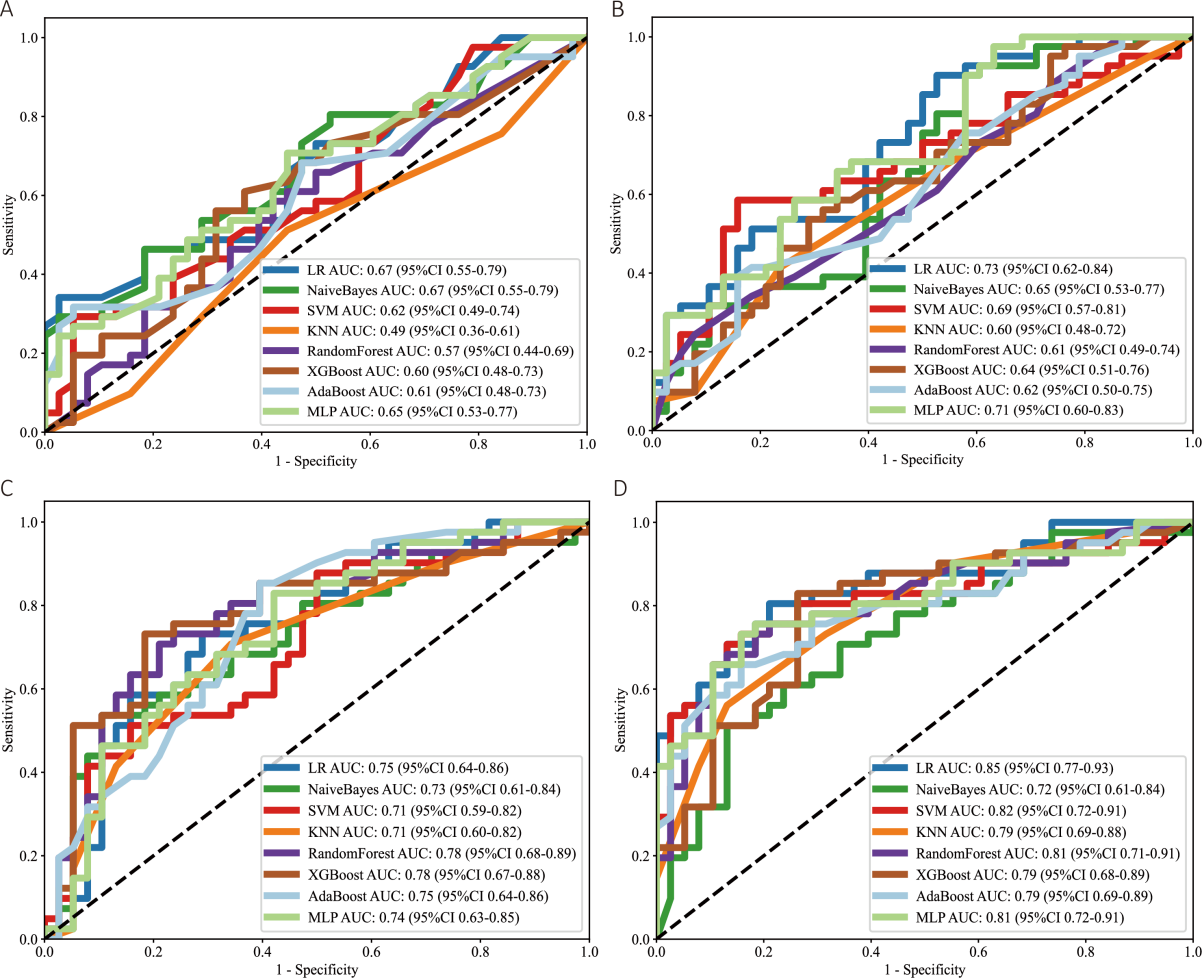
Comparison of predictive performance for the four models in the validation cohort using various machine learning algorithms. **(A)** Clinical-Pathology model, **(B)** Immune-Inflammation model, **(C)** Pre-neoadjuvant therapy (Pre-NAT) Deep Learning (DL) model, and **(D)** Post-2nd-cycle-NAT (Post-2nd-NAT) DL model. The receiver operating characteristic (ROC) curves for each model, generated by eight machine learning methods, are displayed. The area under the curve (AUC) values with 95% confidence intervals are annotated for each curve.

Data from the training set were used for model development. The distribution of characteristics between the pCR and non-pCR groups is presented in **Table 2**. The process for constructing each model is as follows. DCE-MRI DL Models: From the pre-treatment and post-2nd-cycle DCE-MRI images, 2,048 deep learning features were respectively extracted using ResNet-101. After applying the feature selection methods described above, 6 pre-NAT DL features and 10 post-2nd-NAT DL features were selected to construct the Pre-NAT DL model and Post-2nd-NAT DL model, respectively. Detailed procedures for DL feature selection are provided in the **Supplementary Material**. Immune-Inflammation Model: Variables showing significant differences in univariate analysis between the pCR and non-pCR groups in the training set, specifically SII_baseline_, dSII, dPLR, and TILs, were utilized to build the immune-inflammation model. Clinical-Pathological Model: Clinical-pathological indicators demonstrating statistically significant differences between the pCR and non-pCR groups in the training set, namely ER, PR, HER2, and Ki67, were selected to construct the clinical-pathological model. Combined Model: Variables incorporated in the above models were pooled. Through stepwise regression with the minimum Akaike Information Criterion (AIC = 118.19), the final combined model included clinical features (Ki67, ER, PR, HER2), inflammatory features (dPLR, SII_baseline_, TILs), and deep learning features (Pre_DL_F370, Pre_DL_F719, Pre_DL_F727, Post_DL_F297, Post_DL_F414, Post_DL_F711, Post_DL_F727, Post_DL_F732). The AIC-based selection process is illustrated in **Figure 2**. All models were developed using multiple machine learning algorithms. The performance of these algorithms for each model is summarized in **Figure 3**. Based on the criterion of achieving consistently high AUC performance in the validation cohort, logistic regression was selected as the final algorithm for model construction.

**Table 2:** Differences in characteristics between the pCR and non-pCR groups in the training set.

Characteristics	non-pCR (n = 114)	pCR (n = 69)	P value
Age	50.00 (47.00, 52.00)	50.00 (47.00, 53.00)	0.591
Location			0.806
Left	55 (48.25%)	32 (46.38%)	
Right	59 (51.75%)	37 (53.62%)	
Clinical stage			0.158
I	3 (2.63%)	1 (1.45%)	
II	49 (42.98%)	23 (33.33%)	
III	53 (46.49%)	43 (62.32%)	
IV	9 (7.89)	2 (2.90%)	
ER			<.001
negative	33 (28.95%)	46 (66.67%)	
positive	81 (71.05%)	23 (33.33%)	
PR			<.001
negative	43 (37.72%)	45 (65.22%)	
positive	71 (62.28%)	24 (34.78%)	
HER2			<.001
negative	74 (64.91%)	25 (36.23%)	
positive	40 (35.09%)	44 (63.77%)	
Ki67	40.00 (30.00, 60.00)	60.00 (35.00, 70.00)	0.014
TILs			<.001
Low	44 (38.60%)	9 (13.04%)	
High	70 (61.40%)	60 (86.96%)	
CEA	1.67 (0.93, 2.66)	1.48 (1.02, 2.71)	0.954
CA153	10.26 (7.70, 17.23)	10.80 (7.50, 18.30)	0.943
CA125	14.73 (11.47, 24.63)	13.40 (9.72, 20.19)	0.144
NLR_pre-NAT_	2.35 (1.90, 3.35)	2.15 (1.63, 3.06)	0.058
PLR_pre-NAT_	144.25 (118.78, 187.57)	145.88 (121.81, 191.11)	0.984
LMR_pre-NAT_	5.06 (3.93, 6.67)	5.07 (3.97, 6.75)	0.864
SIRI_pre-NAT_	0.73 (0.50, 1.14)	0.69 (0.46, 1.07)	0.489
SII_pre-NAT_	579.96 (407.15, 943.13)	458.28 (325.11, 724.36)	0.013
PIV_pre-NAT_	165.78 (106.73, 288.39)	145.36 (87.85, 286.12)	0.476
NLR_post-2nd-NAT_	2.40 (1.60, 3.38)	2.62 (1.80, 4.30)	0.176
PLR_post-2nd-NAT_	214.97 (155.24, 299.69)	201.35 (147.69, 295.90)	0.445
LMR_post-2nd-NAT_	3.04 (2.34, 4.75)	3.44 (2.54, 4.75)	0.502
SIRI_post-2nd-NAT_	0.95 (0.56, 1.63)	0.97 (0.64, 1.72)	0.634
SII_post-2nd-NAT_	644.84 (385.71, 1057.73)	681.74 (411.43, 1247.88)	0.595
PIV_post-2nd-NAT_	258.73 (122.02, 497.37)	234.89 (136.86, 499.37)	0.976
dNLR	0.18 (-0.62, 1.21)	0.41 (-0.85, 1.62)	0.725
dPLR	80.07 (8.74, 148.78)	35.60 (-8.45, 89.33)	0.003
dLMR	-1.89 (-2.85, -0.44)	-1.66 (-2.93, -0.31)	0.789
dSIRI	0.22 (-0.26, 0.77)	0.28 (-0.12, 0.96)	0.451
dSII	165.88 (-143.35, 608.14)	7.00 (-282.25, 383.44)	0.026
dPIV	80.52 (-43.83, 266.04)	79.86 (-45.60, 271.18)	0.923

Data are presented as n (%) for categorical variables and median (interquartile range) for continuous variables. P-values were calculated using the Chi-squared test (or Fisher’s exact test) for categorical variables and the Mann-Whitney U test for continuous variables. pCR, pathological complete response; Non-pCR, non-pathological complete response; ER, Estrogen Receptor; PR, Progesterone Receptor; HER2, Human Epidermal Growth Factor Receptor 2; Ki-67, marker of proliferation Ki-67; TILs, Tumor-Infiltrating Lymphocytes; CEA, Carcinoembryonic Antigen; CA153, Carbohydrate Antigen 153; CA125, Carbohydrate Antigen 125; NLR, Neutrophil-to-Lymphocyte Ratio; PLR, Platelet-to-Lymphocyte Ratio; LMR, Lymphocyte-to-Monocyte Ratio; SIRI, Systemic Inflammation Response Index; SII, Systemic Immune-inflammation Index; PIV, Pan-immune-inflammation Value; d, delta (change).

### Visualization analysis of deep learning model

**Figure 4:**
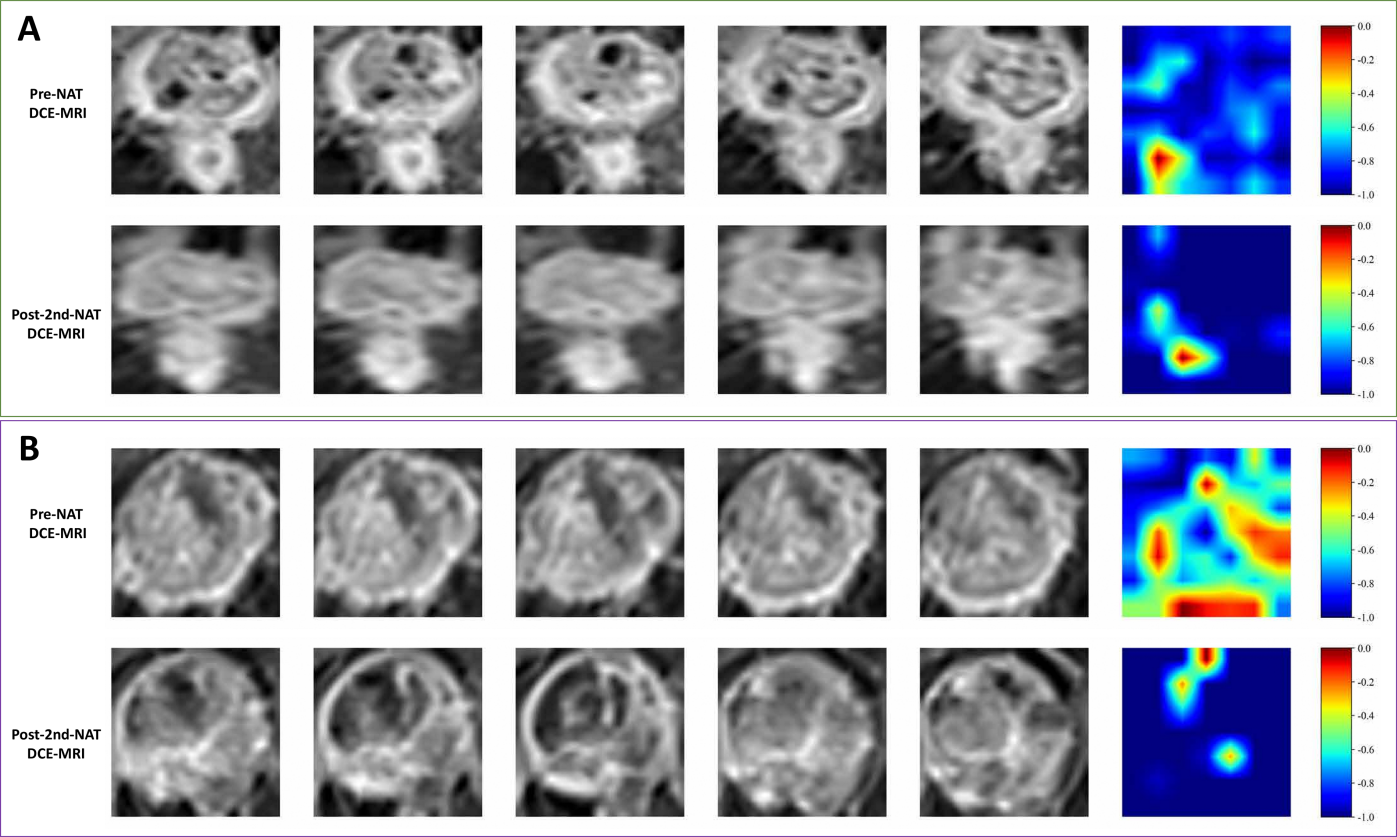
Visualization of deep learning model attention using Gradient-weighted Class Activation Mapping (Grad-CAM). The heatmaps (overlaid in red-yellow) highlight the image regions that most influenced the ResNet-101 model’s prediction of pathological complete response (pCR). **(A)** Representative patient with non-pCR: the spatial distribution and intensity of the model’s focal regions show minimal change between baseline (top) and post-2nd-NAT (bottom). **(B)** Representative patient who achieved pCR: the model’s attention at baseline (top) is concentrated at the enhancing tumor margin. After the second cycle of neoadjuvant therapy (bottom), the extent and intensity of the attention region are markedly reduced.

**Figure 4** presents Grad-CAM heatmaps superimposed on the pre-NAT and post-2nd-NAT DCE-MRI for one representative patient who achieved pCR and one with non-pCR. The heatmaps highlight the spatial regions within and around the tumor that the model attributed the greatest importance to when making its classification decision. In the pCR case, the model’s primary attention was focused on the enhancing tumor margin at baseline. Following the second treatment cycle, these high-attention regions showed a marked reduction in both extent and intensity, aligning with the observed pathological response. In contrast, for the non-pCR case, the spatial distribution and intensity of the model’s focal regions remained largely unchanged between the two time points.

### Model evaluation

**Figure 5:**
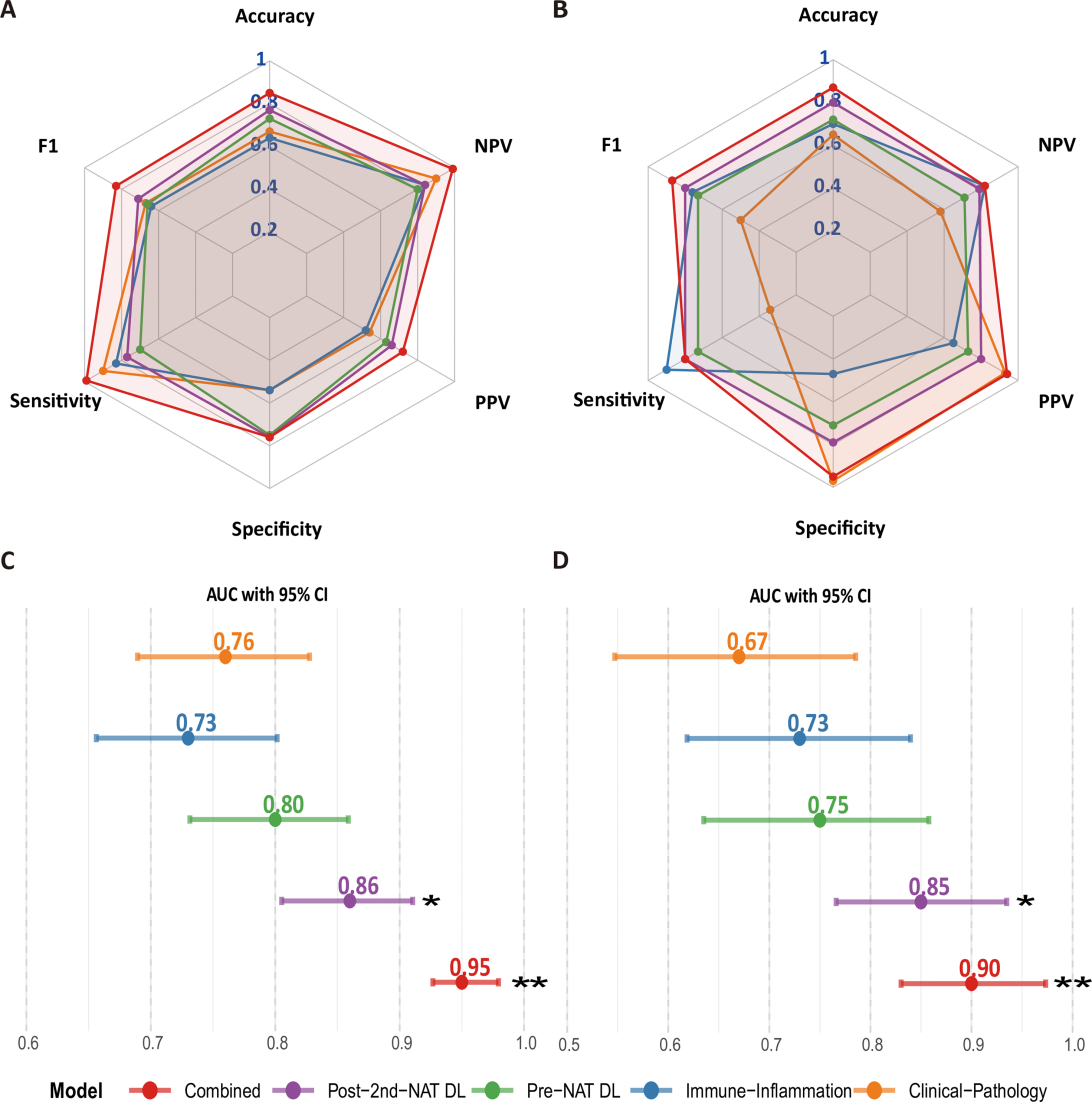
Comparative analysis of model performance across different datasets and evaluation metrics. **(A, B)** Radar charts depicting the diagnostic efficacy of different models in the training and validation sets, respectively. Metrics include Sensitivity, Specificity, Positive Predictive Value (PPV), Negative Predictive Value (NPV), and F1-score. **(C, D)** Horizontal bar charts comparing the Area Under the Curve (AUC) of different models in the training and validation sets, respectively. Error bars represent 95% confidence intervals (CI). **P < 0.01, *P < 0.05 vs. the Clinical-Pathology model (orange).

The comparative diagnostic performance of all models is presented in **Figure 5**. In the training cohort, the combined model demonstrated the best predictive performance, with accuracy, AUC, sensitivity, negative predictive value, and F1-score all exceeding those of other models, reaching 0.85, 0.95, 0.99, 0.99, and 0.83, respectively (**Figure 5A**). The combined model maintained its superior performance in the validation cohort, where it demonstrated the highest discriminative ability, with its accuracy (0.87) and AUC (0.90) being significantly superior to those of any single-modality model (**Figure 5B**). It also demonstrated exceptionally high specificity (0.95) and positive predictive value (0.94), indicating its high reliability in identifying pCR. **Figures 5C, D** show that the AUC values of both the combined model and the Post-2nd-NAT DL model were significantly higher than those of the clinical-pathological model in both the training and validation cohorts (P < 0.05).

**Figure 6:**
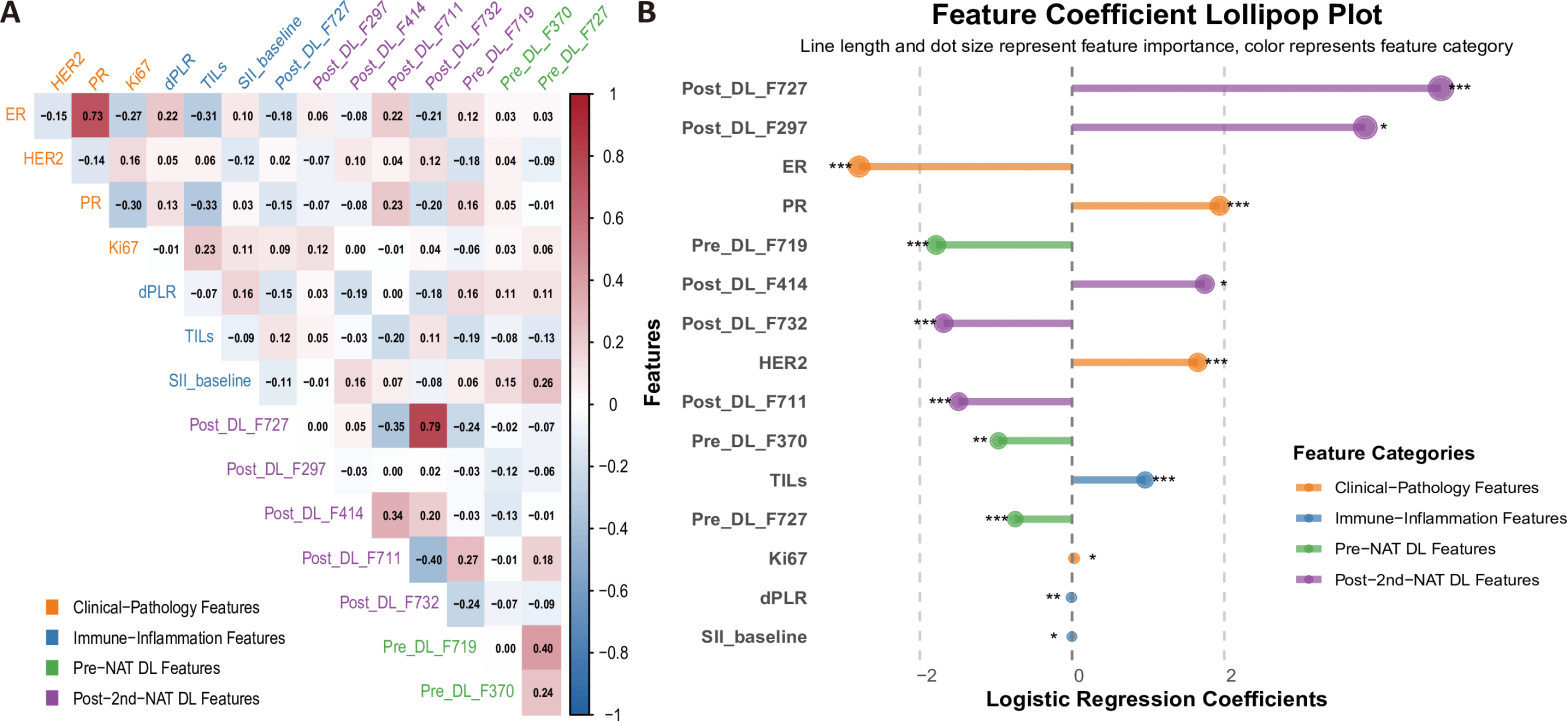
Analysis of variables in the combined model. **(A)** Correlation matrix heatmap of the variables included in the combined model, demonstrating the absence of strong multicollinearity. **(B)** Predictor importance plot (lollipop chart) showing the direction and magnitude of each variable’s association with the treatment response, as determined by the logistic regression coefficients. Feature categories are color-coded. Negative coefficients (red) indicate a negative impact on response, while positive coefficients (blue) indicate a positive impact. ER, estrogen receptor; dPLR, dynamic platelet-to-lymphocyte ratio; SII, systemic immune-inflammation index; TILs, tumor-infiltrating lymphocytes; Pre_DL, pre-treatment deep learning feature; Post_DL, post-treatment (2nd cycle) deep learning feature.

The correlation coefficient plot for the variables incorporated into the combined model is shown in **Figure 6A**, indicating no significant multicollinearity issues. Clinical features (ER), inflammatory features (dPLR, SIIbaseline), and deep learning features (Pre_DL_F370, Pre_DL_F719, Pre_DL_F727, Post_DL_F711, Post_DL_F732) exhibited a strong negative impact on treatment response; whereas clinical features (Ki67, PR, HER2), immune features (TILs), and deep learning features (Post_DL_F297, Post_DL_F414, Post_DL_F727) demonstrated a strong positive impact on treatment response (**Figure 6B**).

## Discussion

Accurately predicting pCR to NAT noninvasively remains a significant challenge in breast cancer management. To address this challenge, our study successfully developed and validated a combined model that incorporates DL features from early longitudinal DCE-MRI, PBI indices, and baseline TILs levels. The combined model demonstrated strong predictive performance in the validation set (AUC = 0.90), significantly outperforming models based on single data modalities. These findings highlight the value of combining longitudinal tumor heterogeneity information with TME-related data for achieving accurate, noninvasive prediction of treatment response during the early cycles of NAT.

Our study confirms the strong potential of DL features based on longitudinal DCE-MRI in capturing dynamic changes in tumor treatment response. Zhou et al. (22) demonstrated that a DL model using CNN on breast masses could predict lymph node metastasis in breast cancer. Xu et al. (23) reported that a 3D ResNet-50 model based on pre-treatment DCE-MRI predicted NAT response in TNBC with an AUC of 0.72. A systematic review on multimodal DL for predicting breast cancer NAT outcomes indicated that CNN was the primary architecture (88.2%), and longitudinal imaging improved prediction compared to baseline imaging alone (24). Consistent with this, our study utilized the ResNet-101 architecture. Furthermore, our study showed that the Post-2nd-NAT DL model (validation cohort AUC = 0.85) demonstrated improved predictive performance compared to the Pre-NAT DL model (validation cohort AUC = 0.75). This finding aligns with the current understanding of tumor heterogeneity and its dynamic evolution under NAT (25). Unlike traditional imaging, which relies heavily on human visual assessment, DL features treat the breast lesion as a subject for pixel-by-pixel quantitative evaluation. This quantitative approach can yield more accurate and reproducible imaging diagnoses than qualitative reasoning (26). Moreover, conventional radiomics and single-time-point imaging often fail to capture the complex spatiotemporal heterogeneity in tumors. In contrast, deep learning methods excel at analyzing longitudinal data. They can effectively decode subtle treatment-induced phenotypic alterations, including intratumoral necrosis, vascular permeability shifts, and cellular density modifications. These changes often serve as early markers of therapeutic response (27). The superior performance of our Post-2nd-NAT DL model over the Pre-NAT DL model further confirms this advantage. Treatment-emergent imaging features provide predictive information that extends beyond static baseline characteristics. This approach facilitates the earlier application of imaging biomarkers to guide precision oncology decisions. Of note, the Grad-CAM heatmaps in our study further indicate that the regions the model focused on for predicting pCR were predominantly located at the tumor margin (tumor-stroma interface), and pronounced longitudinal changes in these regions early during treatment were closely associated with pathological complete response. This suggests that the abstract DL features contributing significantly to the combined model likely encode information related to dynamic alterations in tumor morphology and enhancement patterns induced early by therapy. Although the tumor-stroma interface is known to be a TIL-rich zone, the correlation matrix (**Figure 6A**) showed no strong associations between DL features and TILs. Thus, we hypothesize that these imaging features primarily reflect tumor angiogenesis, vascular permeability, and cellular density rather than direct immune cell infiltration. Future studies with larger cohorts are warranted to further validate the generalizability and biological basis of this spatiotemporal association, and ongoing prospective studies with image-pathology spatial co-registration are expected to clarify the relationship between imaging features and the immune microenvironment.

Our analysis of PBI biomarkers revealed that a higher SII_baseline_ and smaller increases in dPLR and dSII during early treatment were significantly associated with pCR. This finding provides novel insights into the relationship between systemic inflammatory dynamics and response to NAT. Notably, this pattern differs from reports commonly seen in other cancers, such as non-small cell lung cancer (13). This discrepancy may be attributed to the substantial heterogeneity of breast cancer. SII integrates three cell lines closely related to immune inflammation: neutrophils, platelets, and lymphocytes. A higher baseline level might indicate a pre-existing degree of tumor recognition and response by the immune system, potentially providing a foundation for subsequent NAT to elicit a more robust anti-tumor immune effect (28). Significant increases in PLR and SII are often associated with platelet activation, neutrophilia, and lymphocytopenia, changes that typically predict immunosuppression and disease progression (29–31). Consequently, the absence of a substantial increase, or even a trend toward stability or decrease, in these markers during early treatment may indicate that the therapy successfully broke the tumor-induced immunosuppression, preventing further deterioration of the systemic inflammatory state and allowing the immune system to function more effectively (32, 33). Integrating baseline SII with dynamic early-treatment indicators like dPLR and dSII allows for a more sensitive capture of the evolution of systemic immune-inflammatory balance under therapeutic intervention, thereby enabling a more precise assessment of a patient’s potential benefit from NAT. Furthermore, peripheral blood inflammatory markers offer the advantages of easy accessibility, low cost, and high reproducibility (34). As the core component of anti-tumor immunity within the TME, the correlation between baseline TILs levels and pCR has been widely confirmed in breast cancer, especially in triple-negative and HER2-positive subtypes (35, 36). The successful construction of our immune-inflammatory model suggests that a comprehensive assessment framework should encompass both local (TILs in TME) and systemic (peripheral blood inflammatory indices) dimensions, while also considering both baseline status and early treatment dynamics. Such multi-dimensional integrated analysis provides a fresh perspective for understanding the immunobiological mechanisms underlying the heterogeneous efficacy of breast cancer treatments.

Most importantly, the combined model developed through multimodal data integration demonstrated a substantial improvement in predictive performance. By leveraging complementary information from imaging, immunological, and clinical domains, our model potentially overcomes the limitations inherent in single-data-source approaches. For instance, while DL features excel at capturing intratumoral heterogeneity and subtle treatment-induced changes, TILs and inflammatory indicators provide crucial immunological context that helps explain differential treatment responses in radiologically similar tumors. Consistent with this, Janse et al. (37) reported in the LIMA breast MRI trial that tumor subtype remained an important predictor of response to neoadjuvant chemotherapy compared to MR-based DL features. Accordingly, our combined model incorporated key pathological indicators that determine molecular subtypes.

## Limitations

This study has several limitations that should be acknowledged. First, its single-center retrospective design may introduce selection bias. The relatively limited sample size further constrains the generalizability of our findings. Second, all MRI data were acquired using scanners and protocols from a single institution. Although standardization procedures were applied, this limitation necessitates caution. The model’s generalizability requires future validation through prospective, multi-center studies with larger cohorts. Third, our 2.5D input (five consecutive slices) may not fully capture the whole-tumor volumetric heterogeneity. Although five slices covering the tumor core region can capture most of the predictive imaging features, this approach does not equate to a full 3D analysis. Future studies should employ full 3D convolutional networks or more advanced multi-slice fusion strategies to more comprehensively characterize the spatiotemporal heterogeneity of tumors. Fourth, although we employed Grad-CAM to visualize the spatial focus of the deep learning model, the inherent “black-box” nature of deep learning limits full interpretability of the extracted features. The biological underpinnings of these imaging phenotypes remain to be elucidated. Finally, our study focused specifically on predicting pathological complete response. Future research should investigate whether our model can predict long-term outcomes such as disease-free survival or overall survival.

## Conclusion

In summary, this study demonstrates that integrating multi-modal data significantly enhances early prediction of pCR in breast cancer patients receiving NAT. The combined approach incorporates DL features from early-treatment longitudinal DCE-MRI, PBI indices, TILs, and key pathological markers. This multi-scale integration strategy provides a more comprehensive characterization of tumor heterogeneity, holding promise for informing personalized treatment planning in breast cancer patients undergoing NAT.

## Data Availability

The raw data supporting the conclusions of this article will be made available by the authors, without undue reservation.
