# Supplementary material for: Predicting response to neoadjuvant therapy in breast cancer using longitudinal DCE-MRI deep learning integrated with tumor microenvironment data
Source: Front Immunol. 2026 Apr 28;17:1749668. doi: 10.3389/fimmu.2026.1749668 (PMC13161091; doi:10.3389/fimmu.2026.1749668)
Supplement: Supplementary file 1 [file DataSheet1.pdf]

Imaging Acquisition

All breast magnetic resonance imaging (MRI) examinations were performed at Jiangxi Cancer Hospital using two 3.0 T MR systems: a Philips Ingenia (Philips Healthcare, The Netherlands) and a Siemens Prisma (Siemens Healthineers, Germany), both equipped with dedicated 16-channel bilateral breast phased-array coils.

Dynamic contrast-enhanced (DCE) MRI was conducted using a volumetric acquisition technique. On the Philips Ingenia system, the mDIXON sequence was used with the following parameters: repetition time (TR) 3.7 ms, echo time (TE) 1.9 ms, acquisition time 1139.38 ms, slice thickness 2.20 mm, matrix 1024 × 1024, and slice gap 1.10 mm. On the Siemens Prisma system, the DCE examination employed the dyneTHRIVE sequence with the following parameters: TR 4.7 ms, TE 1.7 ms, acquisition time 1971.18 ms, slice thickness 1.50 mm, matrix 1024 × 1024, and slice gap 0.30 mm.

The imaging protocol commenced with a localizer sequence, followed by the DCE-MRI acquisition. A bolus of gadopentetate dimeglumine (Bayer AG, Berlin, Germany) was administered intravenously at a standard dose of 15 mL (0.2 mmol/kg) via an antecubital vein using a power injector (Medrad, Maastricht, The Netherlands) at a flow rate of 2.5 mL/s, followed by a saline flush.

Image Segmentation

All image segmentation procedures were performed in a blinded manner with respect to pathological findings and group assignments. For dynamic contrast-enhanced MRI (DCE-MRI), the third-phase images acquired before and after the second course of neoadjuvant therapy (NAT) were selected and imported in DICOM format into the 3D Slicer software (www.slicer.org). In cases of multifocal or multicentric breast cancer, only the lesions that had been biopsied prior to treatment were selected for analysis. The region of interest (ROI) was delineated slice-by-slice across the tumor on axial images by an attending radiologist (with 10 years of experience) using the software's semi-automatic segmentation tool, thereby generating a volumetric segmentation. All segmented volumes of interest (VOIs) were subsequently reviewed and verified by a second senior radiologist (with 20 years of experience).

Deep Learning Model Construction and Feature Extraction

Model Architecture and Pre-training

The deep learning framework employed in this study is based on the ResNet-101 architecture, a convolutional neural network renowned for its deep structure and exceptional performance in complex visual recognition tasks. The model comprises 101 parameterized layers, integrating convolutional layers, pooling layers, and fully connected layers, with skip connections enabling cross-layer information propagation. This architectural design facilitates identity mapping, effectively alleviating the vanishing gradient problem commonly encountered in deep networks. Prior to its application in this specific task, the model was pre-trained on the ImageNet dataset—a large-scale visual database containing over 14 million images spanning 1,000 object categories. This extensive large-domain pre-training endowed the model with robust visual feature representation capabilities, establishing a solid foundation for subsequent transfer learning in the field of medical imaging.

Global Average Pooling and Feature Vector Generation

A global average pooling (GAP) layer was incorporated as a critical component at the network's terminal stage. Unlike conventional pooling operations that perform localized down-sampling, the GAP layer computes spatial global averages for each feature map from the preceding layer. This design significantly reduces parameter count, effectively mitigating overfitting risks, while preserving comprehensive spatial characteristics through feature-level aggregation. The GAP layer generates a 2048-dimensional feature vector for each input image, encapsulating high-level semantic information essential for image representation. This compact feature representation retains diagnostically relevant patterns while filtering out extraneous details, proving particularly valuable for identifying complex morphological patterns within tumor regions in DCE-MRI images.

This study focuses on tumor region characterization using dynamic contrast-enhanced magnetic resonance imaging. The input to ResNet-101 consisted of five-slice data comprising the largest cross-sectional area of the tumor along with two adjacent slices above and below, enabling 2.5D deep learning feature extraction. All images were resampled to a uniform resolution of 448×448 pixels and center-cropped around the tumor region to enhance relevant signals and minimize background interference. Pixel intensities were normalized to a fixed range of 0-1000 to ensure

training stability and convergence efficiency. Ultimately, using the ResNet-101 model, 2048 deep learning features were extracted from the baseline and post-2nd-cycle treatment DCE-MRI images, respectively.

Deep Learning Feature Selection

Feature selection was conducted separately for each feature group, including pre-neoadjuvant therapy (pre-NAT) DL features and post-2nd-NAT DL features. A total of 2,048 deep learning features were initially extracted from each region of interest (ROI) image. All features underwent standardization prior to subsequent analysis.

LASSO regularization parameter selection: For LASSO regression, we used 10-fold cross-validation to tune the penalty parameter λ. The optimal λ was selected according to the 1-standard error (1-SE) criterion (λ.1se), i.e., the largest λ such that the cross-validated binomial deviance is within one standard error of the minimum. This criterion balances model fit and feature sparsity, enhancing generalizability.

The feature selection process for pre-NAT DL features proceeded as follows: 176 features with p-values <0.05 were initially identified through t-test analysis. Subsequently, Pearson correlation analysis was performed, retaining 38 features with correlation coefficients <0.90. Ultimately, LASSO regression was applied to derive 6 significant features with their corresponding coefficients for model construction.

For post-2nd-NAT DL features, the selection process involved: initial screening via t-test identified 139 features with p-values <0.05, followed by Pearson correlation analysis that retained 70 features with correlation coefficients <0.90. Finally, LASSO regression determined 10 important features with their coefficients for model development.
